# Effectiveness of a Meta-Cognitive Group Intervention for Older Adults with Subjective Cognitive Decline or Mild Cognitive Impairment: The ASPIRE Randomized Controlled Trial

**DOI:** 10.14283/jpad.2024.166

**Published:** 2024-09-25

**Authors:** Shlomit Rotenberg, N. D. Anderson, M. A. Binns, E. R. Skidmore, A. K. Troyer, J. Richardson, F. Xie, E. Nalder, Y. Bar, N. Davids-Brumer, A. Bernick, D. R. Dawson

**Affiliations:** 1 Department of Occupational Science & Occupational Therapy, University of Toronto, Toronto, Canada; 2 Rehabilitation Sciences Institute, University of Toronto, Toronto, Canada; 3 Rotman Research Institute, Baycrest Academy for Research and Education, Toronto, Canada; 4 Departments of Psychology and Medicine (Psychiatry), University of Toronto, Toronto, Canada; 5 Dalla Lana School of Public Health, University of Toronto, Toronto, Canada; 6 School of Health and Rehabilitation Sciences and Department of Occupational Therapy, University of Pittsburgh, Pittsburgh, USA; 7 Neuropsychology and Cognitive Health Program, Baycrest Hospital, Torrance, USA; 8 Department of Psychology, University of Toronto, Toronto, Canada; 9 School of Rehabilitation Science, McMaster University, Hamilton, Canada; 10 Department of Health Research Methods, Evidence, and Impact, McMaster University, Hamilton, Canada; 11 Centre for Health Economics and Policy Analysis (CHEPA), McMaster University, Hamilton, Canada

**Keywords:** Subjective cognitive decline, mild cognitive impairment, randomized controlled trial, functional performance, group intervention

## Abstract

**Background:**

Subjective cognitive decline (SCD) and mild cognitive impairment (MCI) can lead to functional and cognitive decline, increasing dementia risk. There is a pressing need for interventions that prevent this deterioration. The ASPIRE (Adult Strategies Put Into Real-world Environments) intervention was developed to improve performance of daily activities.

**Objectives:**

The primary objective was to determine whether ASPIRE was more effective than a Brain Education control intervention in improving performance and satisfaction with daily life activities that were not specifically trained in the intervention. Secondary objectives were to explore: 1) whether ASPIRE was more effective in improving self-reported health and quality of life, and performance on cognitive tests; and 2) maintenance of change over six-months.

**Design:**

Double-blind, two-armed, parallel randomized controlled trial, with a six-month follow-up period.

**Setting:**

Community based, Greater Toronto Area.

**Participants:**

Two-hundred sixty-four older adults (aged 70.8 ± 6.6 years) with SCD or MCI, randomized to ASPIRE (n=131) or a Brain Education active control arm (n= 133).

**Intervention:**

ASPIRE is a 10-week meta-cognitive group intervention focusing on strategy acquisition and application to improve performance of individualized daily activity identified by each participant as important. It involves setting goals, creating tailored plans, and iteratively modifying these plans with support from the group members and facilitator.

**Measurements:**

Performance of and satisfaction with daily activities was rated on a 10-point Likert scale using the Canadian Occupational Performance Measure (COPM). Secondary outcome were subjective cognition, depression, anxiety, self-efficacy, quality of life, and cognitive tests of memory and executive functions.

**Results:**

Post-intervention, clinically significant improvement of untrained activities (two points or more on the COPM) was found in 32.5% in ASPIRE; and 30.6% in the control arm, with no significant between group differences (Performance: (exp(β̂) =0.96, z=−0.15, p=.879); Satisfaction: (exp(β̂) =0.94, z=−0.29, p=.775). The improvements remained stable over six months in both arms. No significant group effects were found on the secondary outcomes, but improvements were found on subjective cognition and self-efficacy in both arms post intervention.

**Conclusion:**

Both a meta-cognitive strategy approach and an adult learning activity resulted in positive changes in subjective cognition, self efficacy, and, to a certain extent, engagement in daily activities.

## Introduction

**A**s the world’s population ages the prevalence and incidence of dementia of dementia is rising, highlighting the need for effective treatments (1). Given that drug treatments for cognitive impairment in dementia are not highly effective, efforts are focusing on prevention through early intervention in older adults at risk, such as those with subjective cognitive decline (SCD) or mild cognitive impairment (MCI) (2, 3). SCD, characterized by self-perceived cognitive decline not detected by standardised neuropsychological tests (3), is reported is 25% to 50% of older adults aged 65 or older (4). MCI involves cognitive decline beyond normal aging (5, 6) affecting an estimated 17.3% of older adults (7). Both conditions are linked to adverse psychosocial issues like depression, anxiety, and reduced quality of life (8–10).

Despite being generally independent (3, 6), individuals with SCD or MCI may experience difficulty or withdrew from complex activities of daily living (e.g., financial management), or some of their social and leisure activities (11–13). This is concerning because participation in social, leisure, and other community activities is associated with better mood, cognition, physical health, and well-being in older adults (14–17), and was shown to delay the onset of dementia by 3.5 years (16).

Existing non-pharmacological interventions for people with SCD or MCI focus on improving cognitive functions through cognitive training, lifestyle changes (like exercise and diet), and psychoeducation (18–21). However, these interventions often overlook improving non-cognitive outcomes such as subjective cognition and quality of life (19), and there is a lack of interventions addressing daily activities. The ASPIRE (Adult Strategies Put Into Real-world Environments) intervention was developed to address this gap.

ASPIRE is a meta-cognitive group intervention designed to improve the ability of older adults with SCD or MCI to manage daily challenges. It focuses on metacognitive skills like planning, self-monitoring and behavioural regulation, needed to understand functional difficulties and identify effective strategies to overcome them (22). ASPIRE is a modification of the Cognitive Orientation to daily Occupational Performance™ approach (23). Unlike other psychoeducational interventions that concentrate on one type of activity (e.g., physical exercise, diet), ASPIRE targets individualized functional goals and aims to promote self-management of functional challenges in various life areas that are important to each participant.

In our pilot study (n=19), Real World Strategy Training, an early iteration of ASPIRE, resulted in significant improvements in 70% of the trained activities, and 50% of additional activities that were not trained (24). Participants in the experimental arm reported significantly greater improvements in performance of untrained activities compared to those in the control arm, suggesting that they independently applied the strategies they learnt to improve activity performance (24). These results prompted a randomized controlled trial (RCT) to evaluate the effectiveness of ASPIRE.

This RCT examined the effectiveness of ASPIRE in improving performance and satisfaction-with-performance of daily activities, among older adults with SCD or MCI. The primary study objective was to determine whether ASPIRE was more effective than a Brain Education control intervention in improving performance and satisfaction of daily life activities that were not trained in the intervention. Our hypothesis was that ASPIRE participants would report a significantly greater improvement in performance and satisfaction for their untrained activities compared to the control arm. Secondary objectives were: 1) to determine whether ASPIRE was more effective than Brain Education in improving health related quality of life, subjective perception of cognitive functioning, self-reported mood, self-efficacy, anxiety, daily functioning and objective performance on cognitive tests; and 2) to explore maintenance of change over a six-month period in the primary and secondary outcomes.

## Methods

### Study Design

This was a double-blind, two-armed, parallel RCT. Participants were randomly assigned to either the ASPIRE experimental arm or the Brain Education active control arm. Data were collected before randomization (baseline), immediately post-intervention, and three- and six-months post intervention. A study protocol was developed a-priori, and was executed with two changes. First, we expanded the inclusion criteria to include individuals with MCI, not just SCD as originally planned, to enhance recruitment. This decision was based on the recognition that individuals with SCD and MCI face similar functional challenges (12). Additionally, we added a few self-report questionnaires to the secondary outcomes. The study was approved by the Baycrest Health Sciences Research Ethics Board and was registered on ClinicalTrials.gov (NCT03495037). Participants provided written informed consent before enrollment.

### Participants

Participants were recruited through advertising in the community (e.g., local papers, primary physician’s offices, coffee shops), talks at libraries, community centres and senior centres, and from the Rotman Research Institute volunteer database. Participants were eligible if they: 1) met criteria for SCD or MCI as described subsequently; 2) were community-dwelling; 3) were 60–85 years old; 4) identified at least three daily life activities they wanted to improve; and 5) could read, write and converse in English. Participants were excluded if they reported: 1) current moderate-severe depression, determined as score below 9 on the nine item Patient Health Questionnaire (25); 2) past neurological diagnosis with potential long-term effects (e.g., stroke, Parkinson’s disease); 3) past psychiatric related hospitalization; 4) current substance abuse; and/or 5) currently receiving chemotherapy.

Participants’ classification as having SCD or MCI (inclusion criterion a) was determined by two neuropsychologists through a consensus diagnosis, based on the neuropsychological test results (see methods), medical history, and demographic characteristics (e.g., age, education, age of acquiring the English language). Participants were included if they confirm cognitive issues by endorsing at least one of the following questions: “Do you feel that you have problems with your memory or cognition?” or “Do you feel that your memory has become worse?” Participants were classified as having SCD if they did not score below 1.5 standard deviations of age- and education-corrected norms on more than one neuropsychological test within a cognitive domain (i.e., memory or executive function). Participants who scored 1.5 standard deviations below the norms, or more, were classified as either MCI or possible dementia. The latter were excluded. Due to COVID-19 restrictions, eligibility of participants tested after March 13, 2020, was completed using online CANTAB cognitive tests (26).

### Randomization and blinding

Assessors were trained research assistants, unaware of intervention contents and participant arm allocation. Study participants were masked to the classification of their intervention arm, and were unaware of the content delivered in the other arm. Eligible participants were randomly assigned to the experimental or control arm in their preferred community/senior centre, with a 1:1 ratio, using sealed envelopes (27). A computer-generated randomization list was created by MB who was not involved in testing or delivering the intervention. The list was organized in cohorts of 12, with two 6-person groups, one per arm in each cohort. A research assistant placed printouts with arm allocation in sealed, sequentially numbered, opaque envelopes. The research coordinator (YB or NDB) then assigned participants to arms, in order of baseline assessment completion, by opening the next envelope in sequence. To prevent information sharing between the two arms, individuals in partnered relationships (11 pairs) were assigned to the same arm using one envelope. We allowed only one pair per cohort to limit group sizes to a maximum of seven participants.

### Intervention setting

The experimental intervention (ASPIRE) and the active control (Brain Education) each comprised 10 weekly sessions: eight 2-hour group sessions, and two 45-minute individual sessions. An additional booster session was held two months after the intervention. Interventions were delivered in eight community and senior centres in the Greater Toronto Area. The facilitators followed a session-by-session manual to ensure consistency. Participants received a binder containing a schedule, contact details, weekly presentation materials, and worksheets. To mitigate the influence of physical location, seasonal effects or facilitation style, sessions for both arms were conducted concurrently at each site, on different days and/or times, and by the same facilitator. We conducted 46 groups (23 per arm) between March 2018 and August 2020. Follow-up data collection ended in February 2021. A registered occupational therapist (AB) facilitated 40 groups, and the remaining six were delivered by other health professionals (SR, NDB, and YB). Due to the COVID-19 pandemic, five groups (three ASPIRE, two Brain Education) transitioned from in-person to online delivery via video conferencing part way through the intervention, and completed a range of one to eight sessions online. Additionally, six groups (three per arm) were conducted entirely online from May to August 2020, with technological support provided to minimize attrition.

### ASPIRE intervention

ASPIRE participants took part in a strategy acquisition process, working iteratively to improve their performance of the daily activities they had identified for improvement. ASPIRE included three overlapping phases:

1. Acquisition phase (weeks 1–7): Participants were introduced to:
  - Goal-Plan-Do-Check, a global strategy designed to compensate for executive dysfunction. It involves setting SMART (specific, measurable, attainable, realistic, timely) goals, developing actionable plans, and monitoring their implementation and progress towards goal attainment. The facilitator worked with participants on three activities, with goals and plans tailored to individual needs, preferences, and resources. Participants reported on their progress and refined their plans through a problem-solving process supported by group members and the facilitator;
  - Task Specific Strategies (TSSs): Strategies specific to a task or situation, identified by participants and incorporated into the plans. TTSs included mental strategies (e.g., self talk); external compensation (e.g., digital reminders); self-regulation strategies; and task modification (e.g., breaking down task steps).
  - The Strategy Toolbox: An iterative list of TSSs discussed during the intervention and documented in writing by group members. Participants were encouraged to use it as a resource when making plans.
  - Education on the effects of cognitive changes, particularly executive functions, on daily life and self-management, to highlight the need for the global strategy; and on the relationship between activity engagement and healthy aging, to motivate goal attainment.
2. Generalization phase (weeks 5–8): This phase aimed to foster independent management of new and ongoing challenges by helping participants transfer their strategy use skills. Participants were encouraged to apply the global strategy to manage other (untrained) daily activities using TSSs. They could share their experiences, but the facilitator did not provide mediation. Additionally, knowledge on healthy lifestyle behaviors (e.g., exercise, diet, new learning, social and leisure activities) was included to motivate participants toward new goals using the strategies they learned.
3. Maintenance phase (weeks 8–10, and booster session): Discussions focused on identifying barriers and facilitators to sustaining strategy use post-intervention. Participants devised maintenance plans to continue using the global and task-specific strategies (e.g., accessing social support, using reminders). In the final two sessions, they shared updates on their maintenance plans with the group.

To enhance participant autonomy and ownership, the facilitator used guided discovery (23), promoting active learning using a series of sequential, hierarchical questions or guiding statements that lead the participants to discover and articulate a new concept, principle or action. The facilitator also used verbally mediated performance analysis (23), an analytic process of identifying performance difficulties through detailed verbal debriefings.

Fidelity was ensured through review of session videos by team members other than the facilitator, checking for: use of the global cognitive strategy (Goal-Plan-Do-Check) throughout session; collaborative verbally mediated performance analysis; use of guided discovery; verbal expression of awareness of goals by participants; participants engagement with facilitator in discussion regarding their goals and plans throughout session; encouragement of collaboration among group members in discussing goal progress.

### Brain Education active control intervention

The Brain Education intervention included lecture-based classes, using materials developed by Levine et al. (28). Topics covered brain structure, cognitive functions (attention, language, memory, executive functions), neurological conditions, neuroimaging techniques, and age-related cognitive changes. Classes included interactive exercises to enhance engagement and understanding. Homework assignments of Sudoku and word searches were provided to match the engagement of the experimental group outside sessions. The facilitator avoided discussing applying learned content to daily life or strategies for enhancing daily functioning.

### Measurements

#### Demographics and sample characteristics

Demographic information was collected using a self-report questionnaire. Participants’ cognitive status at baseline was characterized using the Montreal Cognitive Assessment (29) and the Wechsler Adult Intelligence Scale III (WAIS-III) vocabulary subset (30).

#### Primary outcome measure – the Canadian Occupational Performance Measure

The primary outcome, performance of untrained activities, was measured using the Canadian Occupation Performance Measure (COPM), a semi-structured interview administered by a health professional (YB, NDB, SR, or AB) at baseline. Participants identified important daily activities they were not performing satisfactorily and rated their importance from 1 (not important) to 10 (extremely important). They then rated their performance and satisfaction with these activities on a 10-point Likert scale, where higher scores indicated better performance and satisfaction. Activities rated eight or higher on performance were excluded, as this indicates high performance levels, rendering the maximum performance score a seven. The COPM is feasible and valid as a rehabilitation outcome among older adults, with adequate content and construct validity, and moderate responsiveness to change (31, 32).

In the ASPIRE arm, three activities identified via the COPM were classified as ‘trained’ and discussed in the sessions: one chosen by the participant in the first individual session, and two others selected randomly. All other activities were ‘untrained’. In the Brain Education control arm, no activities were trained. Untrained activities were not discussed in either arm. As per the COPM manual, a two-point improvement in COPM ratings constitutes a clinically significant change (33). The primary outcome was the proportion of untrained activities that improved by at least two points in the ASPIRE arm compared to the control arm, assessed immediately post-intervention.

#### Secondary outcomes - self-reported health and well-being

We used self-report questionnaires of health and well-being. The Multifactorial Memory Questionnaire (MMQ), measures memory satisfaction, memory ability (mistakes in everyday life), and memory strategy-use (34). The Behavioural Rating Inventory of Executive Functions – Adult version (BRIEF-A) measures emotional, behavioral, and metacognitive aspects of executive functions in adults and older adults (35). The General Self-Efficacy Scale (GSE) measures self-efficacy hypothesised to predict coping with daily problems (36). The Revised Life Orientation Test (LOT-R) measures optimism or pessimism about the future (37). The Geriatric Depression Scale (GDS) (38) and the Geriatric Anxiety Inventory (GAI) (39) measure depressive or anxiety symptoms, respectively, specifically in older adults. The Medical Outcomes Study 36-Item Short-Form Health Survey (SF-36) measures physical and mental health-related quality of life in eight subscales (40). Finally, the Late-Life Function and Disability Instrument (LLFDI) measures three sub-scales of daily functioning: 1) difficulty in performing discrete tasks like dressing or walking (functional); 2) frequency of disability across activities (disability frequency); and 3) level of limitations across activities (disability limitation) (41).

#### Secondary outcomes - cognition

Cognitive functioning was measured with a series of standardized neuropsychological assessments of memory and executive functions: Hopkins Verbal Learning Test-Revised (HVLT-R) immediate and delayed recall (42); Brief Visuospatial Memory Test-Revised (BVMT-R) immediate and delayed recall (43); WAIS-III, Digit Symbol and Digit Span Tests (30); and the Delis–Kaplan Executive Function System (D-KEFS), Trail Making, Colour Word Interference, Tower and Verbal Fluency sub-Tests (44). Raw scores were transformed into standard Z-scores, reflecting the number of standard deviations below or above the mean raw score. The Z-scores were summed by cognitive domains, into a memory composite score, and an executive functioning composite scores.

### Procedures

We measured the outcomes at four time points: baseline, post intervention, and three- and six-months post intervention. The three-month follow-up assessment included only the COPM and some of the self-report questionnaires, to decrease assessment burden. The COPM was completed in-person, except for the three-month follow-up COPM, which was collected by telephone. Self-report questionnaires were completed on paper in-person or online using REDCap electronic data capture tools (45). All data collection past March 2020 (due to the COVID 19 pandemic) was done online or over the telephone. Because the neuropsychological assessments could only be administered in-person, these data are available for 17 of the 23 cohorts post intervention, and for 13 cohorts at the 6-month follow-up.

### Sample size

Target sample size was calculated based on the primary outcome measure. In our pilot study (24), the experimental arm showed a large effect of treatment immediately post-intervention (exp(β̂) =2.5, i.e., the odds of improving on untrained activities was 2.5 times higher in the experimental arm than the control arm). We elected to power this RCT to detect at least a doubling of the odds, to mitigate the possibility that the true treatment effect is smaller than that estimated from the pilot data. To achieve 80% power to detect an effect of this magnitude with a random effect model we required 237 participants. We planned to over sample by 15% to account for possible attrition and to recruit 272 participants (n=136 per arm).

### Statistical analysis

Between group differences on demographic variables, the number of activities identified on the COPM, and COPM baseline ratings are presented using Cohen’s d and Cramer’s V effect size estimates for continuous and categorical variables, respectively. For the primary objective, we used linear mixed effects regression with a binary outcome and random intercept for participant to model changes in the primary outcome. Binary variables were derived for the COPM ratings, indicating whether the participant’s performance or satisfaction score was two or more points higher than its baseline score. For each identified activity, two binary variables (one each for performance and satisfaction) were calculated for the three assessment time points: immediately post-training and three- and six-months later. The intercept was interpreted as the odds of clinically relevant improvement over the intervention period, with four covariates also included in the model: 1) baseline COPM ratings of performance or satisfaction; 2) COPM importance ratings at baseline, standardised using mean centering; 3) time (in months) post intervention {0 = immediately post intervention, 3, 6} indicating change over time after the intervention; and 4) treatment arm, entered as {−½, ½}. A cross-product (interaction) ‘time × arm’ term was subsequently added to the model, to explore whether trajectory of change from post-treatment was different between the two arms, as a potential indication of inability to maintain long-term change. We report the calculated proportion and 95% confidence interval for the improvement in performance and satisfaction. To account for individual differences in the number of identified activities, we calculated an adjusted proportion of change in COPM ratings using mean centering, whereby the sample mean was subtracted from every value. We interpreted the multiplicative effect of each of the four covariates on the odds of performance or satisfaction improvement as exp(β̂) for unit increases of the explanatory variables. Participants who did not provide any COPM ratings post intervention were excluded from this analysis.

We performed two post-hoc analyses. First, we used data from the ASPIRE arm only, and added a contrast coefficient of trained vs untrained activities to the model, to explore whether the performance of trained activities improved more than untrained activities across the three time-points. Second, to explore whether the interventions results differed between participants with SCD and those with MCI, we calculated the linear mixed effects model for change in COPM performance ratings, separately for the two sub-groups. Additionally, sensitivity to potential influence of the COVID 19 pandemic on the primary outcome was examined by repeating the analysis while excluding measurements obtained after the implementation of the first pandemic lockdown in Toronto (March 13, 2020).

We ran similar mixed effects regression models for the secondary outcomes with normal distribution. A random intercept was included to account for individual differences. The outcome measures were adjusted using mean centering. Because these are secondary outcome measures, we used the relative magnitude of the regression test statistics (t-test) to describe patterns of change and did not use p values to determine significance. We report change both during the intervention period (intercept and treatment arm) and after the intervention period (time, and treatment arm by time interaction). A t-test statistic value higher than ±2 was considered a substantive change. We calculated Cohen’s effect size index (f) by dividing the square of the t-statistic by the degrees of freedom estimated using Satterthwaite’s approximation effect sizes by dividing the ratio of the variance due to the effect by the error variance for the effect (46, 47). Effect size of 0.10, 0.25 and 0.40 were considered small, medium and large, respectively (47).

## Results

### Participant flow and sample characteristics

**Figure 1:**
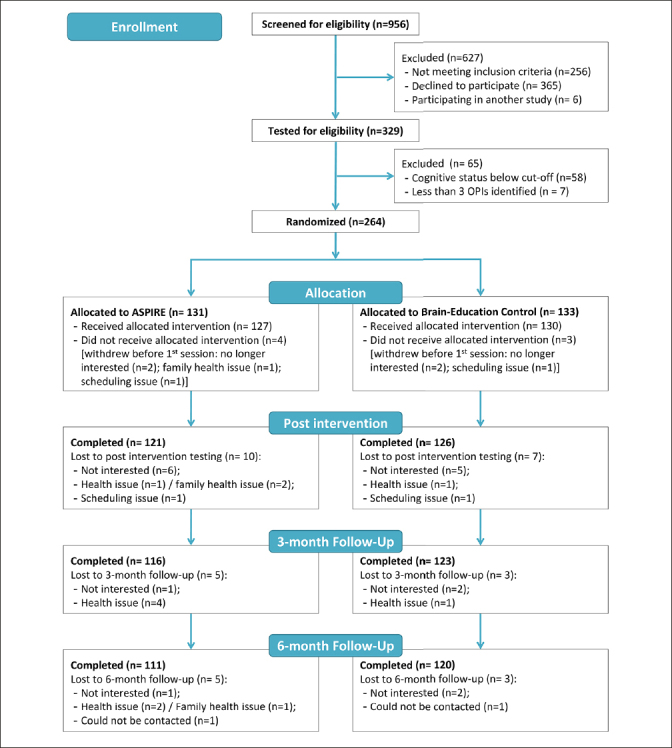
CONSORT diagram

Two-hundred sixty-four people enrolled in the study, and were randomized to ASPIRE (n=131) or Brain Education (n= 133). Figure 1 presents the CONSORT diagram. The COVID-19 pandemic prevented us from reaching the target of N= 272. However, attrition rates were lower than expected, with only 17 withdrawals before post-assessment. The 247 enrolled postintervention exceeded the sample size required according to our power calculations (n= 237). An additional four participants were not included in the COPM analyses because they did not have COPM ratings for untrained activities post intervention. One control arm participant did not provide COPM ratings post-intervention, and three ASPIRE participants had identified only three activities, which were all trained. The 243 participants included in the per-protocol analysis did not differ from the 21 who were not included on number of identified activities and the demographic variables (d = −.02–.31; V =.02–.15), but there were small-medium effect sizes for the cognitive measures (MoCA, d = −.44, WAIS; d = −.40) and the proportion of cognitive classification (V= .235), indicating worse cognitive functioning among those not included in the analysis.

The demographic variables are presented in Table 1. The majority (67%) of participants were women, with an average age of 70.8 ± 6.6 years and 16.9 ± 3.0 years of education. The two arms did not differ on demographic variables, cognitive status, depression, COPM ratings at baseline, and overall number of identified activities as indicated by very small between group effect sizes (Table 1). The control arm had more untrained activities, with a large effect size, due to the study design - all activities in the control arm were untrained, while three activities per participant in the ASPIRE arm were trained.

**Table 1:** Demographics characteristics by arm

	**ASPIRE, n=131 Mean (SD)**	**B-Ed, n=133 Mean (SD)**	**Cohen’s d**
Age (years)	70.64 (6.79)	71.03 (6.47)	−.06
Education (years)	16.69 (2.53)	17.05 (3.48)	−.12
MoCA scores	25.15 (2.92)	25.59 (2.64)	−.15
WAIS-III total score*	52.16 (9.89)	52.25 (9.57)	−.01
PHQ-9	3.56 (2.53)	3.34 (2.47)	.09
Number of activities identified	5.76 (1.12)	5.65 (1.12)	.08
COPM importance	8.00 (1.63)	8.19 (1.57)	−.12
COPM performance	4.44 (2.06)	4.57 (2.02)	−.06
COPM satisfaction	3.60 (2.04)	3.64 (2.08)	−.02
Number of untrained OPIs	2.76 (1.12)	5.7 (1.1)	−2.7
	**n (%)**	**n (%)**	**Cramer’s V**
Sex			.07
Male	38 (29.0%)	47 (35.3%)	
Female	93 (71.0%)	86 (64.7%)	
Gender†			.07
Men	38 (29.2%)	47 (35.6%)	
Women	92 (70.8%)	85 (64.4%)	
Cognitive classification*			.07
SCD	82 (70.7%)	88 (75.9%)	
MCI	34 (29.3%)	28 (24.1%)	
Marital Status†			.13
Married/ common law	76 (58.5%)	77 (58.3%)	
Divorced/separated	16 (12.3%)	26 (19.7%)	
Single	20 (15.4%)	18 (13.6%)	
Widowed	18 (13.8%)	11 (8.3%)	
Race†			.16
White/Caucasian	115 (88.5%)	113 (86.3%)	
Black	3 (2.3%)	3 (2.3%)	
Asian	12 (9.2%)	9 (6.9%)	
Other	0 (0.0%)	6 (4.6%)	

B-Ed= Brain Education; SD = standard deviation; MoCA= Montreal Cognitive Assessment; WAIS-III= Wechsler Adult Intelligence Scale III; PHQ-9= Nine item Patient Health Questionnaire; COPM= Canadian Occupational Performance Measure. *Not available for n=32 participants who completed pre-intervention assessment online; †Total is less then n= 264 because 2–3 participants declined to provide this information.

### Primary outcome

In reporting the COPM results, we use the term ‘improvement in COPM ratings’ to indicate clinically relevant improvement of two points or more. The proportion of COPM improvements and the results of the random effects model are presented in Figure 2A and Table 2, respectively.

**Figure 2A:**
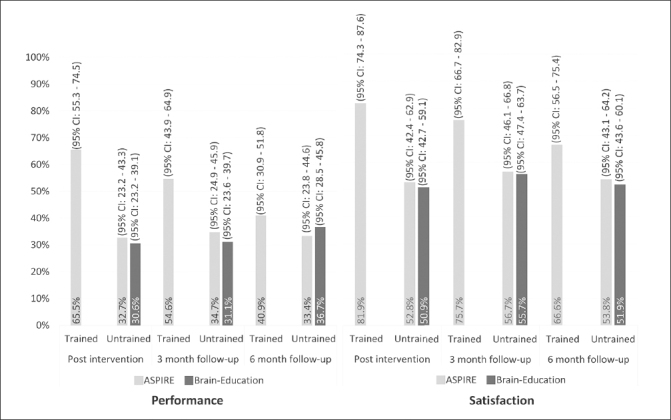
Proportion of COPM improvements in trained and untrained activities

**Table 2:** Linear mixed effects models for COPM ratings improved by two or more points

**Primary outcome: Untrained activities**	Linear mixed effects regression models*			
**Both arms (n=243; 1,074 activities)**	**exp(β̂)**	**z**	**p**	**95% CI**
*COPM Performance*				
Time†	1.03	1.54	.124	0.99 – 1.08
Arm	0.96	−.15	.879	0.58 – 1.60
Baseline performance	0.57	−11.30	<0.001	0.52 – 0.63
Importance	1.19	2.91	.004	1.06 – 1.34
Time by Arm	1.03	0.76	.445	0.95 – 1.13
*COPM Satisfaction*				
Time†	1.01	.34	.737	0.97 – 1.05
Arm	0.94	−.29	.775	0.59 – 1.47
Baseline satisfaction	0.62	−10.80	<0.001	0.57 – 0.68
Importance	1.09	1.56	.118	0.98 – 1.20
Time by Arm	1.00	−0.01	.992	0.92 – 1.09
Post-hoc: Trained and untrained activities ASPIRE arm (n=121; 701 activities)				
*COPM Performance*				
Time†	0.92	−3.04	<0.001	0.88 – 0.97
Activity type (trained / untrained)	1.92	5.09	<0.001	1.50 – 2.48
Baseline performance	0.53	−9.60	<0.001	0.47 – 0.60
Importance	1.13	1.67	0.09	0.98 – 1.31
Time by Activity type	0.92	−3.35	<0.001	0.92 – 1.09
Post-hoc: Untrained activities, SCD group Both arms (n=163; 702 activities)				
*COPM Performance*				
Time†	1.00	−0.08	0.937	0.94 – 1.05
Arm	1.07	0.18	0.855	0.52 – 2.21
Baseline satisfaction	0.54	−9.37	<0.001	0.47 – 0.61
Importance	1.27	3.11	0.002	1.09 – 1.48
Time by Arm	1.00	−0.07	0.947	0.89 – 1.11
Post-hoc: Untrained activities, MCI group Both arms (n=50; 204 activities)				
*COPM Performance*				
Time‡	1.07	1.38	0.167	0.97 – 1.17
Arm	0.49	−1.29	0.198	0.16 – 1.45
Baseline satisfaction	0.61	−5.07	<0.001	0.50 – 0.74
Importance	1.00	−0.01	0.994	0.80 – 1.24
Time by Arm	1.23	2.15	0.031	1.02 – 1.49

95% CI= 95% confidence interval; COPM = Canadian Occupational Performance Measure; *Proportion of improved COPM ratings by 2 points or more, adjusted for between-subject differences in number of identified issues; †Mean number of post intervention assessments per activity: 2.9; ‡Mean number of post intervention assessments per activity: 2.8

### Performance of- and satisfaction with untrained activities

Post intervention, we found an improvement in performance of 32.5% of untrained activities in the ASPIRE arm, and in 30.6% in the Brain Education arm. Satisfaction with untrained activities improved in 52.8% of activities in ASPIRE, and in 50.9% in Brain Education. The random effects model (see Table 2) showed that treatment arm was not significantly associated with improved performance or satisfaction of untrained activities post intervention, meaning that the improvements were similar in both arms. The model also revealed no time effect from post intervention to the two follow-up assessments, indicating maintenance of improvements over time, in both arms. Higher baseline COPM performance and satisfaction ratings were significantly associated with lower probability of improvement in performance and satisfaction ratings, respectively. The importance of the activity was significantly and positively associated with the probability of improving performance, but not satisfaction. The interaction between time and treatment was also not significantly associated with improved performance or satisfaction of untrained activities post intervention. A sensitivity analysis for the main outcome excluding data collected after the first pandemic lockdown (n= 128; 730 activities) did not alter the directions and significance of the findings, with no significant group effect found (Performance: exp(β̂) = 1.03, z= −0.76, p= .445; Satisfaction: exp(β̂) =1.00, z= −0.01, p= .992).

#### Post-hoc analysis: Performance of trained vs untrained activities in ASPIRE arm

The random effects model used on COPM ratings from only the ASPIRE arm showed that trained items were almost twice as likely to have improved than untrained items (OR=1.92; Table 2). We found a significant time effect, indicating that the performance of trained activities declined from post intervention to the two follow-up assessments. Additionally, we found a significant time by activity-type interaction, indicating that the performance of trained activities declined significantly more than that of untrained activities in the six months post intervention. While the improvement of untrained activities remained steady at ∼33% of the activities, the proportion of improved trained activities dropped from 65.5% post intervention to 54.6% and 40.9% three- and six-months after the training ended, respectively.

#### Post-hoc analysis: Sub-group analysis (SCD, MCI) of performance

The random effects model in the sub-group of participants with SCD matched the direction and significance of the findings in the full sample (see Table 2), with no significant group effect on performance of untrained activities post intervention, and no significant group by time effect. The proportion of reported improvement in performance of untrained activities in the SCD sub-group was 36.3% in the ASPIRE arm, and 38.0% in the Brain Education arm. These improvements remained stable over the six-month follow-up period (see Figure 2B). In the MCI sub-group, we also found no significant group effect on performance post intervention (see Table 2), however, the model revealed a significant group by time effect, reflecting differences in the direction of change between the two arms during the follow up period. As presented in Figure 2B, the proportion of improved performance in the MCI-ASPIRE arm increased by ~5% three-months post intervention but decreased by 10% three months later. In the MCI-Brain Education arm, the proportion of improved performance increased by ∼10% at both follow up points. Looking at trained activities in the ASPIRE arm, participants with SCD reported an improvement in performance of 71.2%, and the participants with MCI reported improvement in performance of 52.3% only. As in the full sample, these improvements declined over the six months after the intervention, in both sub-groups (see Figure 2B).

**Figure 2B:**
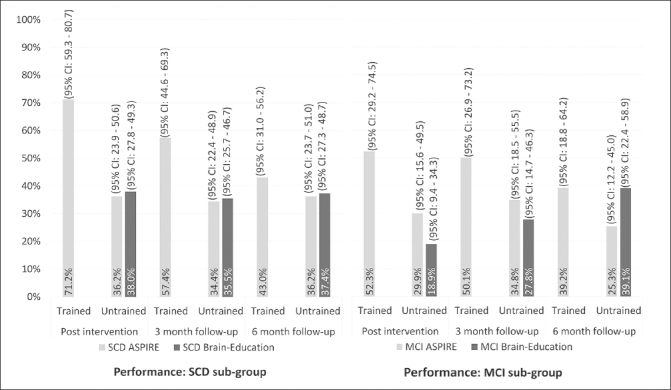
Proportion of COPM improvements in trained and untrained activities

### Secondary outcomes

We found substantive improvements from pre- to post-intervention (intercept t ≥ 2) in subjective cognition (MMQ, BRIEF-A), self-efficacy (GSE), and the SF-36 sub-scale of role limitations due to physical impairment (see Table 3). No differences were found between treatment arms on secondary outcomes, except for a lower frequency of disability (LLFDI) post-intervention in the Brain Education arm compared to the ASPIRE arm. Follow-up assessments showed further improvement in MMQ Satisfaction, while other measures remained steady. Effect sizes of change from pre- to post-intervention were small for most secondary measures, except for large and medium effect sizes in MMQ satisfaction and BRIEF-A, respectively. No effects were found for cognitive functioning composite scores.

**Table 3:** Linear mixed effects regression models for secondary outcome measures

**Self-report Questionaries**	**Pre, Mean (SD)**		**Post, Mean (SD)**		**3m, Mean (SD)**		**6m, Mean (SD)**		**Test statistic (t)**				ES index (f)*
	**ASPIRE**	**B-Ed**	**ASPIRE**	**B-Ed**	**ASPIRE**	**B-Ed**	**ASPIRE**	**B-Ed**	**Intercept (pre-post)**	**Arm**	**Time (post-6m)**	**Arm by Time**	
MMQ-Satisfaction†	36.67 (12.64)	36.08 (11.35)	40.16 (12.84)	41.59 (13.81)	41.37 (13.08)	43.30 (14.11)	42.48 (13.64)	42.98 (13.54)	7.76 ∥	1.49	3.45 ∥	−0.97	0.432 L
MMQ-Ability†	49.11 (10.63)	48.91 (10.40)	51.46 (10.53)	52.04 (12.46)	49.16(10.19)	50.21 (12.09)	53.00 (10.65)	52.08 (13.27)	3.69 ∥	0.65	0.74	−1.46	0.193 S
MMQ-Strategy Use†	35.35 (11.21)	38.35 (10.76)	38.11 (10.45)	39.46 (12.20)	37.75 (10.49)	38.77 (12.77)	36.92 (11.04)	38.57 (12.60)	3.06 ∥	−0.72	−1.41	0.18	0.157 S
BRIEF-A, GEC‡	104.33 (21.05)	105.04 (19.94)	100.98 (19.92)	99.40 (18.79)	NA	NA	99.31 (18.83)	99.97 (20.07)	−5.53 ∥	−1.20	−0.57	1.46	0.283 M
GSE†	30.30 (4.00)	31.19 (3.88)	30.79 (4.51)	32.18 (4.21)	NA	NA	31.22 (4.17)	32.42 (4.32)	3.23 ∥	1.95	1.36	−0.79	0.167 S
LOT-R†	17.65 (4.57)	17.50 (4.77)	17.35 (4.81)	17.96 (4.77)	16.48 (4.98)	17.09 (4.98)	18.22 (4.67)	18.32 (4.76)	0.63	1.47	3.59*	0.01	0.032
GDS‡	2.43 (2.34)	2.27 (2.35)	2.06 (2.22)	2.21 (2.37)	2.61 (2.83)	2.51 (2.82)	2.23 (2.54)	2.40 (2.92)	−1.84	1.17	4.00 ∥	−1.00	0.098
GAI‡	3.81 (4.66)	3.79 (4.48)	3.42 (5.03)	3.40 (4.55)	3.51 (4.99)	3.35 (4.37)	3.08 (4.87)	3.16 (4.64)	−1.83	−0.52	0.39	−0.48	0.096
SF-36 Physical Functioning†	78.17 (19.27)	75.41 (21.36)	78.18 (18.59)	76.55 (22.03)	NA	NA	75.64 (22.25)	73.89 (23.94)	0.46	0.42	−1.52	−0.23	0.022
SF-36 Role-Physical†	65.65 (41.64)	61.28 (40.53)	69.69 (39.05)	70.15 (40.32)	NA	NA	65.70(41.58)	68.88 (41.65)	2.80 ∥	0.13	−0.92	0.58	0.136 S
SF-36 Bodily Pain†	71.26 (19.33)	65.55 (21.64)	70.94 (20.20)	69.29 (21.33)	NA	NA	68.16(21.16)	69.03 (22.32)	1.40	0.68	−0.75	1.37	0.071
SF-36 General Health†	69.02 (16.92)	69.79 (18.09)	69.39 (17.04)	67.89 (20.72)	NA	NA	69.25 (16.49	68.55 (20.43)	−0.80	−1.20	0.31	0.76	0.041
SF-36 Vitality†	57.37 (17.03)	60.26 (18.43)	59.25 (16.88)	62.44 (18.46)	NA	NA	59.30 (19.09)	62.56 (20.36)	1.54	0.67	0.47	0.46	0.078
SF-36 Social Functioning†	82.73 (18.11)	81.20 (19.62)	84.96 (19.42)	82.34 (22.51)	NA	NA	81.49 (21.95)	78.82 (26.03)	1.14	−1.15	−1.71	0.20	0.056
SF-36 Role-Emotional†	74.81 (35.59)	76.44 (34.28)	77.13 (36.03)	75.37 (38.61)	NA	NA	80.95 (34.47)	80.19 (35.01)	0.09	−0.83	2.12 ∥	0.13	0.004
SF-36 Mental Health†	75.48 (14.29)	76.06 (15.81)	76.54 (15.92)	76.22 (16.85)	NA	NA	76.34 (15.57)	75.34 (17.05)	0.68	−0.37	0.03	−0.97	0.034
LLFDI: Dis-frequency‡	62.09 (7.07)	61.75 (7.31)	62.25 (7.01)	60.48 (8.99)	NA	NA	60.89 (7.81)	59.43 (9.49)	−1.30	−2.32 ∥	−3.76 ∥	0.57	0.070
LLFDI: Dis- limitation‡	68.42 (9.77)	67.48 (10.33)	68.81 (9.82)	67.71 (10.81)	NA	NA	67.23 (10.97)	65.40 (12.20)	0.30	−1.03	−3.36 ∥	−0.26	0.016
LLFDI: Functional‡	138.59 (17.51)	135.23 (20.15)	139.73 (15.61)	137.37 (17.79)	NA	NA	140.79 (17.43)	136.87 (19.81)	2.05 ∥	−0.06	0.13	−0.56	0.109 S
**Neuropsycho-logical tests**	**Pre, Z-score range**		**Post, Z-score range**		**3m, Z-score range**		**6m, Z-score range**		**Test statistic (t)**				**ES index (f) a**
	**ASPIRE**	**B-Ed**	**ASPIRE**	**B-Ed**	**ASPIRE**	**B-Ed**	**ASPIRE**	**B-Ed**	**Intercept (pre-post)**	**Arm**	**Time** (post-6m)	Arm by Time	
Executive function composite§	−13.7 – 9.9	−12.2–9.0	−11.8–8.0	−16.9–9.2	NA	NA	−10.1-10.8	−15.4–6.6	0.58	1.47	−1.48	0.42	0.01
Memory composite§	−9.9–9.6	−11.4–7.7	−10.3–10.9	−10.9– 6.5	NA	NA	−7.9-6.6	−12.0–6.9	-0.22	−0.64	−0.12	−0.119	0.06

Pre = pre-intervention (baseline) assessment; SD = standard deviation; Post = post-intervention assessment; 3m = three month follow-up assessment; 6m = six month follow-up assessment; ES = effect size; B-Ed= Brain Education; MMQ = Multifactorial Memory Questionnaire; L = large ES; S = small ES; BRIEF-A, GEC = Behavioural Rating Inventory of Executive Functions – Adult version, global executive composite; NA = not applicable; M = medium ES; GSE = General Self-Efficacy Scale; LOT-R = Revised Life Orientation Test; GDS = Geriatric Depression Scale; GAI = Geriatric Anxiety Inventory; SF-36 = Medical Outcomes Study 36-Item Short-Form Health composite index; LLFDI = Late-Life Function & Disability Instrument; Dis- = Disability; *ES calculated for difference between pre- and post-intervention; †higher score reflects better functioning; ‡higher score reflects worse functioning; §Neuropsychological testing performed on partial sample due to COVID-19 restrictions on in-person testing, n at pre = 232; n at post = 176; n at 6-month follow-up = 123; ∥Substantive change, determined as t-test statistic ≥ ±2

## Discussion

The primary aim of this RCT was to determine if ASPIRE, a meta-cognitive group intervention, was more effective than the Brain Education control in improving performance of untrained daily activities. Our hypothesis was not supported, as there was no difference in the proportion of improved performance of untrained activities between arms post intervention. Performance improved in 30–36% of activities and satisfaction in 53–56% across both arms and time points. In the ASPIRE arm, nearly two-thirds of trained activities improved, but this improvement was only partially maintained over time. Our hypotheses for the secondary outcomes were also not supported, as no group effects were found except for self-efficacy, which improved more in the control arm. Both arms showed small to large improvements postintervention in subjective memory, executive functions, self-efficacy, and one quality of life sub-scale.

### Primary outcome – performance and satisfaction on untrained activities

Performance of untrained activities improved for about one-third of activities in both intervention arms. The COPM interview, performed at baseline in both arms, may have raised participants’ motivation to change their involvement in discussed activities. Reflective motivation, the thought processes of identifying a desired behaviour, planning, and evaluating actions, is essential for behavior change when motivation is not automatic (48). Discussing challenges during the COPM interview may have sparked reflective motivation, leading to performance improvements in both arms. This is supported by literature showing that interviews about physical exercise prompted older adults to reflect on, and address barriers (49). The apparent contribution of the COPM interview to improved performance suggests that helping clients identify activities they consider important is a valuable first step in improving daily functioning of older adults.

While motivation is key for pursuing healthy behaviors, it alone is often insufficient. The Capability, Opportunity, and Motivation framework of Behavior (COM-B) suggests that reflective motivation interacts with an individual’s physical and psychological capabilities, as well as external opportunities such as social support, accessibility, and community resources, to influence behavior (48). For instance, better self-perceived capabilities were significantly associated with increased physical activity during the pandemic, while motivation was not (50). In older adults, both motivation and confidence in ability to make effective changes increase the likelihood of making lifestyle changes (51). This may explain why only about one-third of untrained activities improved through reflective motivation as we suggest. Improved performance likely occurred only when participants also had the necessary capabilities and access to required resources.

Satisfaction ratings of untrained activities postintervention mirrored the trend of performance ratings but were about 20% higher. Our pilot study of ASPIRE and other studies with older adults found similar results (24, 52). Satisfaction with performance in meaningful activities is likely related to the intrinsic value and positive emotions associated with these activities (53). Furthermore, it is possible that as performance improved, participants unwittingly adjusted their expectations about the activity, helping them avoid stress when they did not reach the maximum performance level. This process in known as reframing, an emotion-focused coping strategy that involves changing one’s perception of a situation to lessen its negative emotional effects (54).

### Baseline ratings associated with improved performance and satisfaction

In both study arms, activities with lower initial performance and satisfaction ratings were more likely to show improvement. Activities with high baseline ratings, such as a COPM performance rating of seven—the highest in our study—had limited potential for further improvement. Given that individuals are naturally driven towards activities that fulfill their psychological need for competence (55), it is also possible that participants were less motivated to work on activities with high baseline ratings, that indicate a high level of competence and contentment. Additionally, activities rated as more important at baseline were also more likely to show improved performance, likely motivated by alignment with participants’ life choices, their values and commitments (55).

### Improved performance in trained vs untrained activities

Our post-hoc analysis in the ASPIRE arm showed greater improvement in the performance of trained activities immediately after the intervention compared to untrained activities. Based on the COM-B model (48), we suggest that ASPIRE improved performance of trained activities by enhancing reflective motivation beyond the initial COPM interview; developing participants’ meta-cognitive problem-solving skills (capabilities); providing direct social support from the group and facilitator; and assisting in accessing community resources (opportunities).

Contrary to our hypothesis, ASPIRE participants did not report greater improvement in untrained activities compared to the control arm. This suggests that the meta-cognitive skills and strategies acquired were not generalised beyond the trained activities. Although ASPIRE aimed to reduce the cognitive load associated with strategy use (22) by introducing structured strategies such as the Goal-Plan-Do-Check global strategy and the strategy toolbox, it appears that these tools were not sufficient for independent strategy use. Another potential barrier to generalization was the wide variety of target activities identified by participants, which differed significantly in physical, spatial, and social characteristics, as well as complexity (12). This diversity required participants to independently create new task-specific strategies, complicating the generalization of strategies from trained to untrained activities.

### Maintenance of improvement over time

The proportion of improved performance in trained activities decreased from 66% post-intervention to 41% after six months. In contrast, the improvements in untrained activities remained stable over time in both study arms. Six months post-intervention, the proportion of improved trained and untrained activities was similar. It is possible that the sustained improvement in trained activities was primarily due to enhanced motivation, similar to the improvement untrained activities. Maintaining these improvements required minimal additional effort. Other trained activities likely improved through the use of the global problem-solving strategy and TSSs, with assistance from the group members and facilitator. The decline in performance of these activities likely reflects the limited use of strategies needed to maintain the improvements. Ongoing strategy use is effortful and time-consuming (56), and difficult to sustain without the emotional support and advice from the group and facilitator. Social support is key to sustain older adults’ involvement in healthy lifestyle behaviours (57, 58). Future research should investigate whether extended programming for older adults with SCD or MCI, that provides ongoing support, such as periodical booster sessions, could effectively sustain improved performance over time.

### Sub-group analysis (SCD, MCI) of performance of untrained activities

The sub-group analysis that explored performance separately for participants with SCD and participants with MCI showed that ASPIRE was no more effective than the Brain Education control in improving performance of untrained activities post intervention in either sub-group. In the six months after the intervention, surprisingly, performance of untrained activities in the Brain-Education arm of the MCI sub-group improved substantially, while performance in the ASPIRE arm improved in the fist three months, but then declined, reflected in a significant group by time effect. These trajectories are difficult to explain. Similar unexplained results of improvements in untrained activities during the follow-up period in the control arm only were found in our pilot study with SCD participants (24). We will continue to explore possible reasons for changes in performance of untrained activities post intervention in a future qualitative study.

ASPIRE participants with SCD reported a higher proportion of improvement of trained activities than those with MCI, suggesting that they benefitted more from direct training. This does not seem to be explained by differences in the types of activities between participants with SCD or MCI, because a qualitative analysis of activities identified by 109 of our participants showed similar activities identified in both sub-groups, most frequently physical exercise, social-leisure activities, and time or task management, in both groups (12). The baseline importance and performance ratings were also similar in the two sub-groups and do not explain the differences in improvement of trained activities. It is likely that the meta-cognitive process involved in ASPIRE was more challenging for those with MCI due to their cognitive impairment, and they benefitted less from it. The cognitive load involved in ASPIRE may have further led to decreased motivation and adoption of strategy use. This warrants further investigation, because this meta-cognitive approach had been shown to be appropriate for older adults with MCI (59).

### Secondary outcomes – self report questionnaires

Contrary to our hypothesis, ASPIRE did not show larger effects than the Brain Education control arm on secondary self-report outcome measures. We found similar improvements on self-report measures in both arms. Given the small effect sizes, analyzing the specific mechanisms of change within each intervention is not sensible. Generally, we suggest that the group context and social interaction with peers may explain the improvements observed in both arms. We suggest that the small improvements on self-report measures are a result of a pleasant social interaction with peers with similar problems, as well as a meaningful and challenging learning experience provided by both interventions (15, 60).

Notably, both interventions resulted in improved subjective cognition over time. Specifically, we found a large effect size for memory satisfaction, and a medium effect for executive functions in everyday life. Subjective cognition is the core issue in SCD, yet recent systematic reviews found little to no treatment effects of non-pharmacological interventions on subjective cognition (19, 61). In ASPIRE, enhanced knowledge and strategy use likely improved subjective cognition by providing a sense of control over cognitive impacts on daily life. In the Brain Education arm, improved subjective cognition may be due to participation in a cognitively stimulating and challenging activity, shown to improve health in aging (15). It is also possible that subjective cognition improved because the group setting provided an experience of universality with other people with similar cognitive issues, thereby normalizing the experience of cognitive decline.

### Study limitations

This study had several limitations. The sample was predominantly white (over 85%), highly educated (mean ≈17 years), and mostly women (67%). Results may differ in a more diverse sample. The COVID-19 pandemic limited in-person data collection and recruitment, and data collected after March 2020 may have been biased by social distancing policies. However, sensitivity analysis showed no major difference between pre-pandemic and all data results.

Using the COPM to measure performance may have contributed to improvements of untrained activities. Finally, the 21 participants excluded from the primary outcome analysis were more cognitively impaired, which might have affected the results.

## Conclusions

Contrary to our hypothesis, ASPIRE was no more effective than a Brain Education intervention in improving performance of untrained daily activities; nor in enhancing subjective cognition or general self-efficacy. Despite the RCT null results, both interventions resulted in improved performance of approximately one-third of untrained activities and enhanced subjective cognition and self-efficacy. Understanding the mechanisms behind these improvements will help develop a stronger multicomponent intervention to improve the health and well-being of older adults with SCD or MCI. Future protocol development will aim to integrate meta-cognitive strategies with meaningful and engaging learning activities to maximize their combined benefits. Additionally, we will explore ways to ensure sustained improvements in trained activities and better generalization of skills to untrained activities, perhaps by involving family members or other sources of social support, and/or offering ongoing peer-led support.

## Notes

*Ethical standards:* The study procedures were approved by the Institutional Research Ethics Board, and all participants provided written informed consent.
